# The Assessment of TikTok as a Source of Quality Health Information on Human Papillomavirus: A Content Analysis

**DOI:** 10.7759/cureus.75419

**Published:** 2024-12-09

**Authors:** Rhoda E Etta, Abdulhammed O Babatunde, Praise O Okunlola, Oluwatomisin K Akanbi, Kehinde J Adegoroye, Rofiat A Adepoju, Samuel T Tundealao

**Affiliations:** 1 Medicine and Surgery, University of Ibadan, Ibadan, NGA; 2 Dentistry, University of Ibadan, Ibadan, NGA; 3 Physiotherapy, University of Ibadan, Ibadan, NGA; 4 Biostatistics and Epidemiology, Rutgers University, Piscataway, USA; 5 Cancer Health Equity Center of Excellence, Rutgers Cancer Institute of New Jersey, New Brunswick, USA

**Keywords:** health information, hpv, human papillomavirus, reliability, social media, tiktok

## Abstract

Background

Various studies have evaluated the quality of health-related information on TikTok (ByteDance Ltd., Beijing, China), including topics such as COVID-19, diabetes, varicoceles, bladder cancer, colorectal cancer, and others. However, there is a paucity of data on studies that examined TikTok as a source of quality health information on human papillomavirus (HPV). This study, therefore, evaluated the quality of health information on HPV on TikTok.

Methods

The terms “HPV” and “human papillomavirus” were searched on TikTok on a single day in August 2024, and 200 videos were retrieved. Relevant user metrics were collected for each video, including the number of likes, shares, and followers, the video length, and the uploader type. Two independent raters assessed each video regarding the completeness of six types of content (the definition of HPV, symptoms, risk factors, evaluation, management, and outcomes). Then, the two raters independently assessed the quality of information in the videos using the DISCERN instrument.

Results

Sixty-nine videos met inclusion criteria; 11 were created by general users, 44 by healthcare professionals, and 14 by organizations. Videos uploaded by general users and health professionals have a longer duration (p < 0.001) and more likes (p = 0.048) than those uploaded by organizations. More than 60% of the videos contained little or no content on the HPV content assessed. Although the reliability and quality of treatment choices were higher among videos uploaded by healthcare professionals, the overall quality of HPV health information using the DISCERN instrument was “very poor” (24.2 (±6.92)).

Conclusions

The overall quality of HPV videos uploaded on TikTok is very poor and not acceptable, thus failing to satisfy public health needs. Healthcare professionals must enhance their social media presence, produce reliable and substantive material, and collaborate with social media platforms and high-engagement accounts to facilitate users' access to high-quality data. TikTok users must recognize that material regarding HPV may lack medical accuracy and should consistently consult healthcare providers for medical guidance.

## Introduction

The Internet is an essential part of people's daily lives [1], and its accessibility has made about 80% of its users turn to it for health information [1]. Over the past two decades, social media has become a common internet medium for the public to access health information [2]. Social media are web-based applications for social messaging, networking, information creation, and exchange [3]. Examples include X (X Corp., Bastrop, TX), WhatsApp (Meta Platforms, Inc., Menlo Park, CA), Facebook (Meta Platforms, Inc., Menlo Park, CA), and TikTok (ByteDance Ltd., Beijing, China) [3]. Social media offers various benefits to health institutions, health practitioners, and the general public, e.g., users seek advice about their health concerns, lifestyle, and fitness tips, get a second opinion after a doctor's visit, and access trending health news, among other things [4,5].

TikTok is a popular social media application that features user-generated short videos ranging from 15 seconds to 10 minutes, used for sharing information [6]. Launched in 2017 by the Chinese company ByteDance, the application quickly gained attention during the COVID-19 pandemic when social distancing measures were in place. The application is available in over 150 countries and supports 75 languages, boasting 800 million monthly users [6]. Originally known for lip-syncing and comedy, TikTok has expanded to include content related to health, business, food, beauty, and more [6].

Despite the many health benefits, the use of social media also comes with the risk of spreading health misinformation [7]. Health misinformation in the context of public health intervention refers to false or misleading information about health-related topics that can undermine evidence-based practices, hinder effective health communication, and negatively impact public health outcomes [7]. Health information shared on social media can be divided into expertise-based information, shared by health professionals, and experience-based information, shared by laypersons [7]. Although there has been an increase in expertise-based health information on social media [2], non-health professionals still contribute a large share of the health information on social media [8]. This leaves room for health misinformation, which can confuse, reduce trust in healthcare, delay seeking treatment, and inadvertently lead to life-threatening health risks [8].

Human papillomaviruses (HPVs) are nonenveloped, double-stranded DNA viruses belonging to the *Papillomaviridae* family [9]. With over 200 identified types, HPV primarily targets squamous epithelial cells found in the skin and mucosal surfaces, including the anogenital and oropharyngeal regions [9]. These infections can lead to the development of both benign lesions and malignant tumors [9]. Remarkably, it is estimated that approximately 80% of sexually active women and 90% of sexually active men will contract at least one type of HPV during their lifetime [10].

Social media has proven to be an important way of reaching the public with information on HPV and HPV vaccination [11]. About 69% of all TikTok users are between the ages of 13 and 24 years, making it an ideal means of reaching the target population that is a priority for HPV vaccination [11]. This has sparked researchers' interest in TikTok and led to various studies evaluating the quality of health-related information on TikTok, including topics such as COVID-19, diabetes, chronic obstructive pulmonary disease (COPD), bladder cancer, colorectal cancer, and others [12]. However, there is a paucity of data on studies that examined TikTok as a source of quality health information on HPV. This study, therefore, evaluated the quality of HPV-related health information on TikTok.

## Materials and methods

Study design and search strategy

This was a content analysis cross-sectional study conducted in August 2024 using a methodology similar to that used by Basch et al. [13] and Boatman et al. [14]. Using the keywords “human papillomavirus” and “HPV”, the TikTok application was searched, and TikTok's recommended sorting process produced 200 videos. The selection process was done in a day to avoid distortion of video results and order.

Inclusion and exclusion criteria

**Figure 1:**
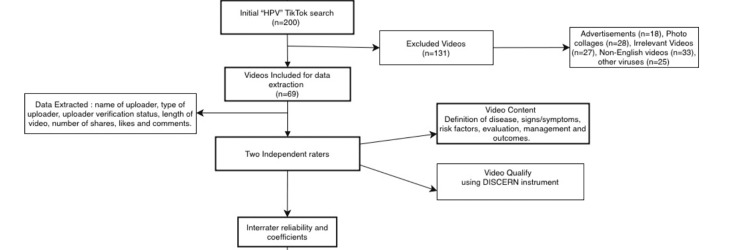
Video extraction process and search results

The videos obtained were sorted based on direct relation to HPV. Excluded videos included advertisements (n = 18), photo collages (n = 28), irrelevant videos not related to HPV (n = 27), videos not in English (n = 33), and videos about other viruses (n = 25), after which we obtained 69 videos, which were then subjected to further data extraction and analysis (Figure 1).

Video content and quality measurement

An assessment tool by Goobie et al. [15] was adopted to measure the quality of the content. Two independent raters scored the videos based on the tool's six content types: the definition of HPV, signs and symptoms, risk factors, evaluation, management, and outcomes. Videos were scored on a five-item scale: 0 points (no content), 0.5 points (little content), 1 point (some content), 1.5 (most content), and 2 points (extensive content).

The quality of information of the videos was measured using the DISCERN instrument, a widely used instrument for assessing the quality of health information [16] and one of the common instruments that can also be applied to video content [17-19]. It consists of 16 questions, with responses based on a five-point scale: 1 = poor to 5 = good. These 16 questions were divided into three sections: section 1 assessed the reliability of the publication (questions 1-8), section 2 evaluated the quality of information on treatment choices (questions 9-15), and section 3 provided an overall rating of the publication (question 16).

Statistical analysis

Cohen's kappa coefficient was used to measure the level of agreement between raters while also considering the possibility of agreement occurring by chance (κ = 0.86, excellent). The characteristics of the videos (length, likes, comments, and shares) were presented using descriptive analysis stratified by the type of uploader and presented as median and interquartile range (IQR) due to the skewness of the data. One-way analysis of variance (ANOVA) (and Bonferroni test) test was used to assess the association between the video’s characteristics and the type of uploader. The quality of the videos was presented in proportion with values for no content, little content, some content, most content, and extensive content for the six content types: the definition of HPV, signs and symptoms, risk factors, evaluation, management, and outcomes. The maximum score for reliability is 40 points, the quality of information on treatment choices is 35 points, the overall video rating is 5 points, and the total DISCERN score is 80 points. From the literature, a DISCERN score of 64-80 is considered excellent, 52-63 good, 41-51 fair, 30-40 poor, and 16-29 very poor [20]. A one-way ANOVA (and Bonferroni test) test was also used to assess the association between the different sections, the overall DISCERN score, and the type of uploader. Analyses were done with "Statistics and Data" (STATA), version 17.0 (Stata Statistical Software, StataCorp LLC, College Station, TX), and the significance level was determined using a two-sided p-value < 0.05.

## Results

Video sources and characteristics

Two primary sources of the videos were identified: individual users (79.7%) and organizational users (20.3%). Among the individual users, we identified two major groups of uploaders: health professionals and general users. Health professionals uploaded more videos (n = 44, 80.0%) than general users (n = 11, 20.0%) (Table 1).

**Table 1:** Description of the video uploaders (n = 69)

Uploader	n (%)	Description
Individual users	55 (79.7%)	These uploaders, who are either health professionals or general users, have no affiliation with any public or private organizations. They included (1) health professionals: individuals who claimed to be health professionals, including doctors, nurses, pharmacists, dentists, health experts, and nutritionists - 44 (80.0%), and (2) general users: individuals who are just active users of the application without claiming profession - 11 (20.0%).
Organizations	14 (20.3%)	Different public or private bodies.

In all the selected videos (n = 69), the shortest video lasted only five seconds, while the longest lasted five minutes and 51 seconds. Overall, the median length of the videos was 51 seconds. Videos uploaded by general users (66 seconds) had a higher median length than those uploaded by health professionals (47 seconds) and organizations (38 seconds) (p < 0.001). The videos in the sample had a combined 921,961 likes and 217,233 comments and were shared 36,728 times. Videos uploaded by general users (148) and health professionals (132) had higher median comments than those uploaded by organizations (51) (p = 0.048). Although not statistically significant, general users and health professional videos also had higher median likes and shares than those uploaded by organizations (Table 2).

**Table 2:** Characteristics of videos across uploaders IQR = interquartile range

Uploaders	Total (n = 69)	General users (n = 11)	Health professionals (n = 44)	Organizations (n = 14)	p-value
	Median (IQR)	Median (IQR)	Median (IQR)	Median (IQR)	
Length of video (seconds)	51 (18, 74)	66 (33, 180)	47 (15, 68)	38 (22, 72)	<0.001
Likes	1,497 (292, 6,444)	4,432 (1,180, 7,195)	1,870 (524, 8,152)	264 (143, 466)	0.668
Comments	123 (47, 259)	148 (68, 464)	132 (49, 271)	51 (33, 67)	0.048
Shares	119 (28, 687)	120 (45, 705)	168 (58, 837)	35 (12, 63)	0.145

Video content quality

The obtained scores from the average scores of the two raters ranged from “no content” (0) to “extensive content” (2). The results showed that more than half of the selected videos contained little content or no content on all six types of content: definition, symptoms, risk factors, evaluation, management, and outcomes. Only approximately 10% of the videos introduced the management of HPV (Table 3).

**Table 3:** Quality of the video’s content

Content	Definition	Symptoms	Risk factors	Evaluation	Management	Outcomes
	n (%)	n (%)	n (%)	n (%)	n (%)	n (%)
No content	35 (50.7)	36 (52.9)	37 (54.4)	38 (55.1)	34 (49.3)	35 (50.7)
Little content	17 (24.6)	16 (23.5)	16 (23.5)	13 (18.8)	25 (36.2)	20 (29.0)
Some content	14 (20.3)	10 (14.7)	10 (14.7)	12 (17.4)	5 (7.3)	9 (13.0)
Most content	1 (1.5)	2 (2.9)	1 (1.5)	3 (4.4)	2 (2.9)	2 (2.9)
Extensive content	2 (2.9)	4 (5.9)	4 (5.9)	3 (4.4)	3 (4.4)	3 (4.4)

Information quality - DISCERN score

The overall DISCERN score was 24.2 (±6.92), which depicts that the quality and accuracy of HPV health information shared via videos were “very poor.” Although not significant, the information shared by health professionals (24.8 (±7.52)) and organizations (24.2 (±7.41)) has better DISCERN (i.e., quality and accuracy) than those shared by general users (21.9 (±1.92)) (p = 0.466). The overall reliability (13.9 (±4.39)) and quality of information about treatment choices (8.4 (±2.63)) were also very poor. The reliability and quality of information about treatment choices are also better in videos uploaded by health professionals and organizations than in videos uploaded by general users (Table 4).

**Table 4:** DISCERN scores of HPV-related TikTok videos by uploader (source) HPV = human papillomavirus; SD = standard deviation

Uploaders	Total	General users	Health professionals	Organizations	p-value
DISCERN scores	Mean (SD)	Mean (SD)	Mean (SD)	Mean (SD)	
Reliability (items 1-8) - 40 points	13.9 (±4.39)	12.6 (±1.80)	14.2 (±4.79)	13.9 (±4.55)	0.586
Treatment choices (items 9-15) - 35 points	8.4 (±2.63)	7.5 (±1.04)	8.6 (±2.92)	8.3 (±2.52)	0.469
Overall rating (item 16) - 5 points	2.0 (±0.86)	1.8 (±0.65)	2.0 (±0.89)	2.0 (±0.96)	0.638
Total (items 1-16) - 80 points	24.2 (±6.92	21.9 (±1.92	24.8 (±7.52)	24.2 (±7.41)	0.466

## Discussion

This study assessed the use of TikTok, a popular social media platform, as a source of health information on HPV. For some users, TikTok has replaced widely used search engines such as Google as a significant source of information [21]. Given the emergence of TikTok, the videos examined in our study received numerous likes and comments and were shared thousands of times, which indicates that TikTok is a promising channel for health communication.

We categorized video uploaders into two primary groups: individual users and organizational users, each encompassing several specialized user types. Individual users comprised general TikTok users, who are merely active participants of the service without a specified occupation, and health professionals, including doctors, nurses, pharmacists, dentists, health specialists, and nutritionists. Organizations consisted of various public or private entities. In this study, healthcare professionals uploaded the majority of HPV videos (80.0%) on TikTok, whereas organizations provided the fewest HPV-related videos. Research has indicated that healthcare practitioners and organizations can leverage social media for efficient health communication and public health advocacy [22]. Our study, however, revealed that healthcare practitioners have actively employed TikTok to disseminate HPV-related health information, although the engagement of health organizations with this burgeoning social media platform has been restricted. This implies that health professionals are the major creators of health-related information relating to HPV on TikTok. This is similar to a study conducted in 2021 by Kong et al. assessing the quality of diabetes-related videos on TikTok, where 69.3% of the contents assessed were by health professionals [22].

This study found that average social media engagements per post were higher for content creators who are general users compared to health professionals, meaning that more people liked, commented, and shared the content on HPV created by general social media users. It was found by Kong et al. that health professionals created less engaging content [22]. General TikTok users frequently produce relatable, interesting, or entertaining content that appeals to a wider audience, hence increasing its likelihood of being widely shared and engaged [23]. Conversely, healthcare professionals may prioritize the dissemination of factual and technical information, which, although precise, may lack appeal or shareability for the broader populace.

The current study revealed that more than 60% of the videos contained little or no content on the HPV content assessed: definition, signs and symptoms, risk factors, evaluation, management, and outcomes. This sharply contrasts with a comparable study that evaluated YouTube (Google Inc., Mountain View, CA) as a source of HPV information, reporting that most videos (81.4%) provided general information regarding HPV and addressed the link between HPV infection and cancer [24]. The disparity in HPV information quality between TikTok and YouTube can be ascribed to the platforms' distinct formats, audience demographics, and content generation dynamics. TikTok's short-form video format, generally restricted to a few seconds or minutes, emphasizes entertainment and brevity, rendering it less suitable for conveying comprehensive instructional material. The user demographic is predominantly younger, frequently interacting with trending, visually captivating, or hilarious content rather than comprehensive informational videos [25]. Conversely, YouTube accommodates longer-form videos, facilitating in-depth conversations on subjects such as HPV, and its viewership encompasses a wider age demographic, including individuals actively pursuing educational or health-related information [25]. Furthermore, YouTube creators frequently generate more structured and researched content, whereas TikTok's algorithm prioritizes swift engagement, thereby diminishing informational depth.

Although the videos uploaded by health professionals had higher reliability compared to general users and organizations, the reliability of the videos was suboptimal. This shows that health professionals are more likely to create content that is more reliable than non-health professionals. This is expected as health professionals have more knowledge of the subject matter. Another study has also reported similar findings by Gurler et al. in 2022 [26]. However, this also shows the potential of social media as a medium for the high risk of misinformation [2]. For instance, despite creating less reliable content, non-health professionals create more engaging content on the subject matter and reach more users per post. Studies have shown that low-quality content achieves more visibility on social media [2]. 

The quality of treatment choices by the video content was generally poor. However, similar to the reliability of video, health professionals had the highest mean score of quality of treatment choices, followed by organizations and general users. Most of the contents focused on the screening and prevention of HPV. This is because HPV is usually asymptomatic but manifests symptoms when it causes diseases such as genital warts, cervical cancer, anal cancer, and oropharyngeal cancer [27]. Hence, treatment choices often target managing these diseases. However, there is a need to improve the quality of treatment choices as social media content influences the choice of healthcare by social media users [28]. Healthcare professionals should focus on creating accurate, evidence-based, and engaging HPV-related video content that emphasizes clear guidance on prevention, screening, and management options tailored to their target audience's level of health literacy. Additionally, incorporating visuals, credible sources, and culturally sensitive messaging can enhance the content's reliability and accessibility, encouraging informed healthcare decisions among viewers.

The "very poor" (24.2 (±6.92)) overall quality of HPV-related health information on TikTok, as assessed by the DISCERN score, can be ascribed to the platform's content creation dynamics and the absence of quality control measures. TikTok's algorithm emphasizes engagement metrics such as views, likes, and shares, frequently privileging entertaining or sensational content above factual and dependable health information [29]. The platform also lacks stringent vetting or moderation of health-related content, hence exacerbating the spread of misinformation [29]. The conjunction of TikTok's brief video format restricts the capacity to thoroughly address intricate health subjects such as HPV, hence exacerbating the prevalence of substandard information. A study that assessed TikTok as a source of quality information for varicoceles also found that the overall quality of the videos ranged from poor to very poor [30]. On the other hand, a study that assessed the quality of diabetes-related TikTok found it to be fair, with a DISCERN score of 47.7 (±7.71) [22].

This study's findings have some implications for public health practice, research, and policy. Healthcare experts and organizations should emphasize the development of entertaining and accurate HPV-related content specifically designed for TikTok's distinct audience to reconcile dependability with engagement. The research findings necessitate exploring creative techniques to augment the dissemination and influence of high-quality health information on social media platforms. Targeted educational initiatives utilizing TikTok's popularity among youth could be crucial in enhancing increasing HPV vaccination rates and awareness of HPV-associated risks. Additionally, the findings highlight the broader challenge of health misinformation on social media platforms. To address this, healthcare organizations should implement strategies such as debunking common HPV myths through engaging content, providing clear action steps for vaccination and prevention, and using TikTok’s algorithm to boost the visibility of trustworthy videos. From a policy perspective, strengthening partnerships between public health authorities and social media platforms is vital for establishing content moderation frameworks that not only curtail misinformation but also promote the dissemination of accurate, high-quality health information. These initiatives collectively optimize TikTok’s potential as a health communication tool while safeguarding public health against misinformation's adverse effects.

Our study had several limitations. Alternative hashtags concerning HPV could include or omit essential information regarding the subject. Although the selection process used might have reduced bias, with the chosen keywords, some relevant HPV-related content might have been missed, especially if creators use non-standard or colloquial language to discuss HPV. The videos analyzed in our study were exclusively in English, and the research did not encompass HPV-related information in other languages. The health professionals' status was self-reported, perhaps lacking accuracy or verifiability. As with all cross-sectional studies, the results are not generalizable. This particularly applies to social media research, as content is always evolving and can affect the most prevalent hashtags, video counts, comment numbers, etc. The powerful algorithm of TikTok tailors video appearances based on users' past behaviors, which can significantly skew research results, thereby potentially undermining the study's findings. However, our study utilized a newly created TikTok account with no past user behavior. We conducted the study and search from Nigeria, and since TikTok's algorithm often prioritizes regionally trending videos, this could have influenced the selection and characteristics of the videos assessed. A potential bias may exist in the subjective evaluation of content and the video selection process. The TikTok account utilized for video search and its geographical context may have influenced the videos obtained through the search mechanism. This may have affected the diversity and comprehensiveness of the videos incorporated in the study. This study did not include the geography/location of the uploaders. Future research efforts on this topic should incorporate this important measure of social determinants of health. Despite these limitations, our study is the first to analyze the reliability and quality of HPV-related videos and not restrict to only HPV vaccinations on TikTok.

## Conclusions

Although there are relatively fewer healthcare professionals on TikTok due to the entertainment-focused algorithm and audience, the HPV-related videos from healthcare experts were more reliable. However, the overall quality of HPV videos disseminated by both healthcare and non-healthcare professionals on TikTok is very poor and unacceptable, thus failing to satisfy public health needs. Healthcare professionals must enhance their social media presence, produce reliable and substantive material, and collaborate with social media platforms and high-engagement accounts to facilitate users' access to high-quality data. TikTok users must recognize that material regarding HPV may lack medical accuracy and should consistently consult healthcare providers for medical guidance. We recommend that TikTok implement a policy for posting health statements akin to cigarette packaging; however, instead of a warning, it should provide a source reference for information verification or a reminder that the content reflects the uploader's personal perspective and may lack verification, thereby enhancing audience awareness. This study also highlights the role of public health campaigns in effectively harnessing social media.
